# Safety and efficacy of an engineered hepatotropic AAV gene therapy for ornithine transcarbamylase deficiency in cynomolgus monkeys

**DOI:** 10.1016/j.omtm.2021.09.005

**Published:** 2021-09-14

**Authors:** Julien Baruteau, Sharon C. Cunningham, Berna Seker Yilmaz, Dany P. Perocheau, Simon Eaglestone, Derek Burke, Adrian J. Thrasher, Simon N. Waddington, Leszek Lisowski, Ian E. Alexander, Paul Gissen

**Affiliations:** 1 Genetics and Genomic Medicine Department, Great Ormond Street Institute of Child Health, University College London, London WC1N 1EH, UK; 2 National Institute of Health Research, Great Ormond Street Biomedical Research Centre, London WC1N 1EH, UK; 3 Metabolic Medicine Department, Great Ormond Street Hospital for Children NHS Foundation Trust, London WC1N 3JH, UK; 4 Gene Therapy Research Unit, Children’s Medical Research Institute and Children’s Hospital at Westmead, Faculty of Medicine and Health, The University of Sydney, Sydney, Australia; 5 Department of Pediatric Metabolic Medicine, Mersin University, Mersin 33110, Turkey; 6 Translational Research Office, University College London, London, UK; 7 Enzyme Unit, NIHR BRC, Great Ormond Street Hospital Foundation Trust and UCL Great Ormond Street Institute of Child Health, London, UK; 8 Molecular & Cellular Immunology, Great Ormond Street Institute of Child Health, University College London, London WC1N 1EH, UK; 9 Gene Transfer Technology Group, Institute for Women’s Health, University College London, 86-96 Chenies Mews, London, UK; 10 MRC Antiviral Gene Therapy Research Unit, Faculty of Health Sciences, University of the Witswatersrand, Johannesburg, South Africa; 11 Translational Vectorology Unit, Children’s Medical Research Institute, The University of Sydney, Westmead, NSW, Australia; 12 Military Institute of Medicine, Laboratory of Molecular Oncology and Innovative Therapies, Warsaw, Poland; 13 Discipline of Child and Adolescent Health, Sydney Medical School, Faculty of Medicine and Health, The University of Sydney, Westmead, NSW 2145, Australia

**Keywords:** adeno-associated virus, ornithine transcarbamylase deficiency, engineered capsid, biodistribution, liver, AAV, AAVLK03, non-human primates, cynomolgus macaque, immune response

## Abstract

X-linked inherited ornithine transcarbamylase deficiency (OTCD) is the most common disorder affecting the liver-based urea cycle, a pathway enabling detoxification of nitrogen waste and endogenous arginine biosynthesis. Patients develop acute hyperammonemia leading to neurological sequelae or death despite the best-accepted therapy based on ammonia scavengers and protein-restricted diet. Liver transplantation is curative but associated with procedure-related complications and lifelong immunosuppression. Adeno-associated viral (AAV) vectors have demonstrated safety and clinical benefits in a rapidly growing number of clinical trials for inherited metabolic liver diseases. Engineered AAV capsids have shown promising enhanced liver tropism. Here, we conducted a good-laboratory practice-compliant investigational new drug-enabling study to assess the safety of intravenous liver-tropic AAVLK03 gene transfer of a human codon-optimized *OTC* gene. Juvenile cynomolgus monkeys received vehicle and a low and high dose of vector (2 × 10^12^ and 2 × 10^13^ vector genome (vg)/kg, respectively) and were monitored for 26 weeks for in-life safety with sequential liver biopsies at 1 and 13 weeks post-vector administration. Upon completion of monitoring, animals were euthanized to study vector biodistribution, immune responses, and histopathology. The product was well tolerated with no adverse clinical events, predominant hepatic biodistribution, and sustained supra-physiological OTC overexpression. This study supports the clinical deployment of intravenous AAVLK03 for severe OTCD.

## Graphical abstract

**Figure:**
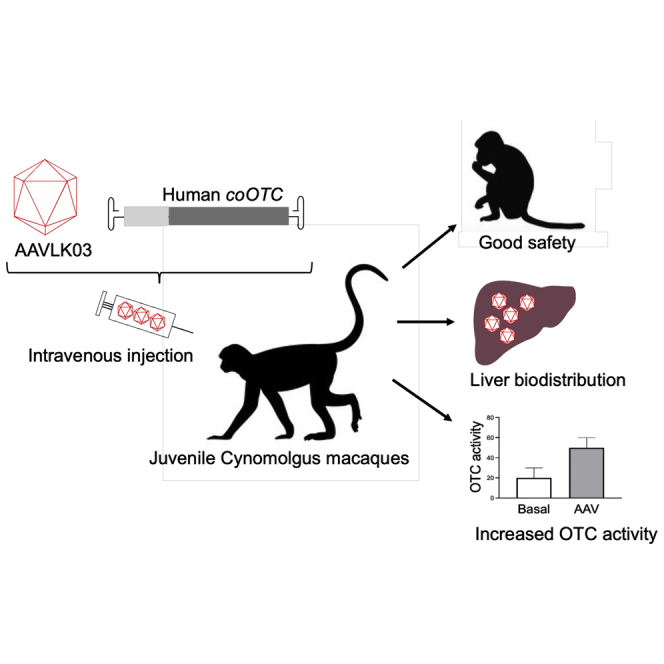

## Teaser

A preclinical study of the AAVLK03 vector encoding the human ornithine transcarbamylase (OTC) transgene administered intravenously in juvenile cynomolgus macaques shows no safety concerns, preferential liver biodistribution, and persistent supraphysiological increase of liver OTC activity. These findings support a phase I/II clinical trial in pediatric OTC-deficient patients.

## Introduction

The urea cycle is a liver-based metabolic pathway enabling the detoxification of ammonia, a neurotoxic product of nitrogen waste produced by protein catabolism, and arginine biosynthesis. Ornithine transcarbamylase deficiency (OTCD) is an X-linked disorder with an estimated incidence between 1:17,000 and 1:60,000 live births and the most common urea cycle defect.^1^^,^^2^ Patients present a hyperammonemic crisis causing acute neurological symptoms, which can lead to coma and death if untreated. The standard of care relies on a protein-restricted diet, daily ammonia scavengers, and arginine supplementation. This treatment does not prevent acute hyperammonemia triggered by intercurrent illness or fasting, which can cause severe neurological sequelae illustrating high unmet needs. Up to now, liver transplantation is the only curative therapy; however, it is limited by organ shortage, requires lifelong immunosuppression, and is associated with significant morbidity.^3^^,^^4^

OTCD has been recognized for many years as an appealing disease candidate for gene therapy. A historical clinical trial with an adenoviral vector encoding the *OTC* gene caused the death of one late-onset OTCD patient receiving a high-dose (HD) vector due to a severe immune response syndrome triggered by innate immunity against the vector capsid.^5^^,^^6^ This fatal outcome somewhat compromised the development of gene therapy for diseases such as OTCD until non-pathogenic adeno-associated viruses (AAVs) were successfully delivered for other liver monogenic disorders such as hemophilia A and B.^7^, ^8^, ^9^ AAV vectors have demonstrated long-lasting transgene expression and disease-modifying efficacy in hemophilia B.^10^ The safety profile is satisfactory, predominantly triggering a CD8 T cell-mediated immune response causing asymptomatic transaminitis well controlled by an oral course of corticosteroids.^10^ However, very HDs (>5 × 10^13^ vector genome [vg]/kg) have induced a severe immune response syndrome with complement activation in large animal models and in clinical trials with fatal outcomes highlighting AAV dose-limited toxicity.^11^, ^12^, ^13^ Using a wild-type AAV8 capsid, a phase 1/2 trial recruiting late-onset adult OTCD patients sponsored by Ultragenyx has shown partial or complete response in 6 out of 9 patients at doses of 2 × 10^12^ to 2 × 10^13^ vg/kg.^14^

To improve hepatocyte transduction, engineered AAV capsids with enhanced liver tropism have been generated.^15^^,^^16^ AAVLK03 is an engineered capsid that shares 97.7% homology of the *cap* sequence and 98.9% homology of the amino acid sequence with the wild-type hepatotropic AAV serotype AAV3B.^15^ AAVLK03 transduces human hepatocytes 1 log better than AAV8 in chimera mouse-human livers and is more resistant to neutralizing antibodies (Nabs) compared to wild-type capsids.^15^^,^^17^^,^^18^ Interestingly, the prevalence of anti-AAVLK03 Nabs is particularly low in pediatric populations.^19^ This capsid is currently in clinical development for adults with hemophilia A sponsored by Spark Therapeutics (ClinicalTrials.gov: NCT03003533).^20^ Moreover, one patient was injected with an AAVLK03 vector containing the *MMUT* gene in a phase I/II open-label clinical trial for pediatric patients with methylmalonic acidemia (SUNRISE study; ClinicalTrials.gov: NCT04581785).^21^

Treating pediatric OTCD patients is clinically relevant, as these patients have a more severe phenotype and are at risk of developing hyperammonemic decompensation with fatal outcome or causing severe lifelong neurological sequelae. AAV vectors deliver a transgene cassette, which persists mainly as a non-integrating nuclear episome that is not effectively transmitted during mitosis. The liver growth in young patients represents a challenge, as transgene loss and dilution may limit the long-term efficacy of AAV gene therapy. To advance AAV gene therapy for pediatric OTCD patients, we have designed an AAVLK03 vector encoding the human codon-optimized *OTC* gene. We performed a good laboratory practice-compliant toxicology study using juvenile cynomolgus macaques to assess vector safety, biodistribution, and immune responses.

## Results

### Study design

**Figure 1:**
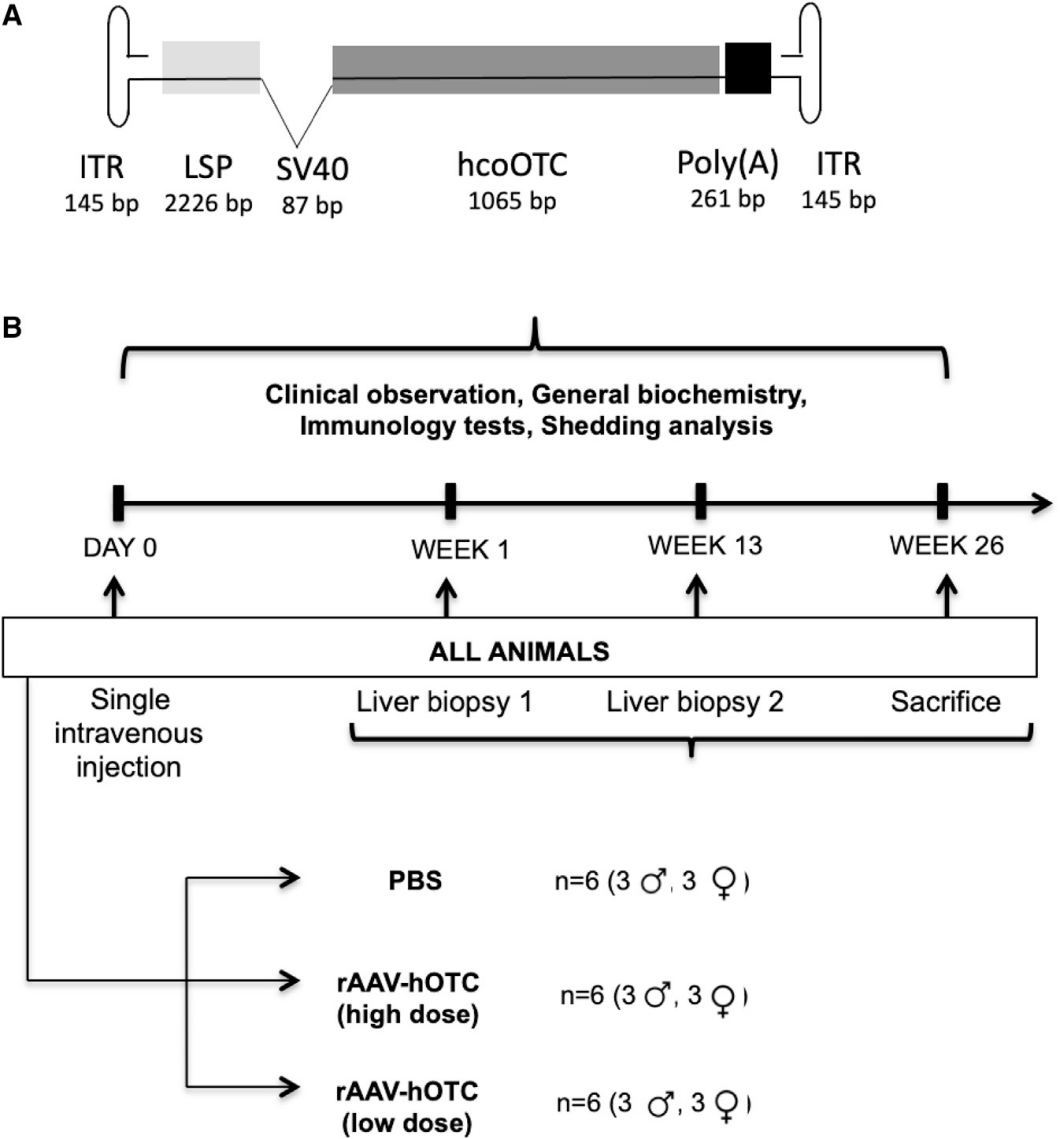
Experimental design for preclinical assessment of AAVLK03.LSP.hOTC (A) Vector schematic of AAVLK03.hOTC. The liver-specific promoter (LSP) contains an ornithine transcarbamylase (OTC) enhancer and the human α1 anti-trypsin promoter. (B) Experimental design of the toxicity study. Three investigational groups of juvenile cynomolgus macaques were monitored for 26 weeks after a single intravenous injection of the AAVLK03.hOTC vector before sacrifice and organ collection. Over a 26-week monitoring period, biofluids were collected at regular intervals, and needle liver biopsies were performed at 1 and 13 weeks post-vector administration. hcoOTC, human codon-optimized OTC transgene; ITR, inverted terminal repeat; PBS, phosphate-buffered saline; rAAV, recombinant adeno-associated viral vector.

Three gender-matched groups of six cynomolgus macaques, aged 18 months, seronegative for Nabs against AAVLK03 received a single peripheral intravenous injection of vehicle, low dose (LD; 2 × 10^12^ vg/kg) or HD (2 × 10^13^ vg/kg) of AAVLK03.human OTC (hOTC) vector encoding the hOTC transgene (Figure 1A). Over a 26-week monitoring period, biofluids were collected at regular intervals, and needle liver biopsies were performed at 1 and 13 weeks post-administration. The animals were sacrificed after 26 weeks, and organs were collected for analysis. No immunosuppression was given (Figure 1B).

For dose selection, we considered the efficacy outcome of a former clinical trial targeting adult hemophilia B patients with a single intravenous injection at 2 × 10^12^ vg/kg with an AAV8-derived vector.^10^ For safety reason, no liver biopsy was performed in this trial. We assumed that a mild increase of plasma factor IX from <1% to 6% should correspond to a low percentage (likely <5%) of hepatocyte transduction. This conclusion is in accordance with the percentage of human hepatocytes transduced by AAV8 vectors in Fah^-/-^/Rag2^-/^ /Il2rg^-/-^ (FRG) mice.^15^ As the AAV-LK03 capsid is expected to have at least 10 times better transduction efficacy in human hepatocytes compared to AAV8 based on *in vivo* studies in FRG mice,^15^^,^^22^ a LD of 6 × 10^11^ vg/kg AAV-LK03 vector would be equivalent to 6 × 10^12^ vg/kg AAV8 vector and transduce around 10%–15% hepatocytes, which is deemed sufficient to observe already an improvement of the phenotype according to experiments in the sparse fur/abnormal skin and hair (*Spf*^*ash*^*)* mice and estimated enzymatic threshold expected to normalize the phenotype in urea-cycle defects in humans.^23^

### AAV vector quality control (QC) testing

The AAVLK03.hOTC appeared in a clear, colorless solution with no visible particles by visual inspection. A bioburden test by direct plating did not identify any bacterial growth (<2 colony formation units/mL), and bacterial endotoxin assessed by Limulus amebocyte lysate (LAL) kinetic chromogenic method was not detected (<0.05 endotoxin units (EU)/mL). The empty-to-full AAV capsid ratio was assessed by optical density by A260/A280 ratio spectrophotometry and found to be at 1.5, in line with acceptable criterion for the manufacturer (>1.3). The SDS-PAGE/silver stain test assessing purity was comparable to the reference. The titer measured by qPCR was 2.66 × 10^13^ vg/mL.

### In-life safety parameters

All animals survived until their scheduled necropsy at 26 weeks. The vector-treated animals had no treatment-related clinical changes identified on general observations, temperature, and heart-rate measurements. Food consumption was adjusted according to body weight, and all animals maintained their weight gain during the study. Mild bruising was noted at the injection site lasting 4 days or less after vector administration. Ophthalmological evaluations were satisfactory. Electrocardiogram was unremarkable in all animals.

### Laboratory parameters

Clinical laboratory parameters, including hematology, coagulation, and biochemistry, did not differ between vehicle- and vector-treated animals at 4, 13, and 26 weeks post-administration (Tables S1–S4). Urine analysis revealed significantly higher relative urine gravity in HD compared to LD, the control group, and pretest values. This difference was considered as not clinically significant.

### Histopathology

An extensive tissue collection listed in Table 1 was performed at necropsy 26 weeks after treatment. All tissue samples had a normal macroscopic appearance. There was no difference in organ weight between AAVLK03.hOTC-treated and control animals. Histologic evaluation did not reveal any noticeable pathological lesions in the AAVLK03.hOTC-treated or control monkeys. Specifically, no ischemic, apoptotic, inflammatory, or mitotic changes were seen. The microscopic examination of necropsy tissues also did not reveal treatment-related changes.

**Table 1:** Tissues and organs collected at necropsy

Tissues/organs				
Adrenal glands	aorta	bone (femur and sternum)	bone marrow	brain (medulla/pons, cerebral, and cerebellar cortex)
Epididymides (males)	esophagus	eyes (with optic nerve)	gallbladder	heart
Intestine, large (cecum, colon, and rectum)	intestine, small (duodenum, jejunum, and ileum)	kidneys	lacrymal gland	larynx
Liver	lungs (with bronchi)	lymph nodes (mesenteric, retropharyngeal, and axillar)	mammary gland	ovaries
Pancreas	Peyer’s patches	pituitary gland	prostate gland	salivary glands (mandibular, sublingual, and parotid)
Seminal vesicles (males)	sciatic nerve	skeletal muscle	skin (with subcutaneous tissue)	spinal cord (cervical, midthoracic, and lumbar)
Spleen	stomach	testes (males)	thymus	thyroid glands
Tongue	trachea	ureters	urinary bladder	uterus and cervix (females)
Oviducts (females)	vagina (females)			

### Immune response

#### Humoral immunity

There were no detectable anti-AAVLK03 Nabs in any vector-treated animals before administration. Control animals were either seronegative or seropositive with a low antibody titer (1/5 dilution). No relevant changes in the Nab titers against AAVLK03 were recorded in control animals during the 26-week observation period. Overall, all AAVLK03.hOTC-dosed animals showed a peak of Nab titers in plasma 2 weeks after vector administration, except for female 20 and male 7 from the HD group, for which the peak occurred at weeks 4 and 13 after the administration, respectively.

**Figure 2:**
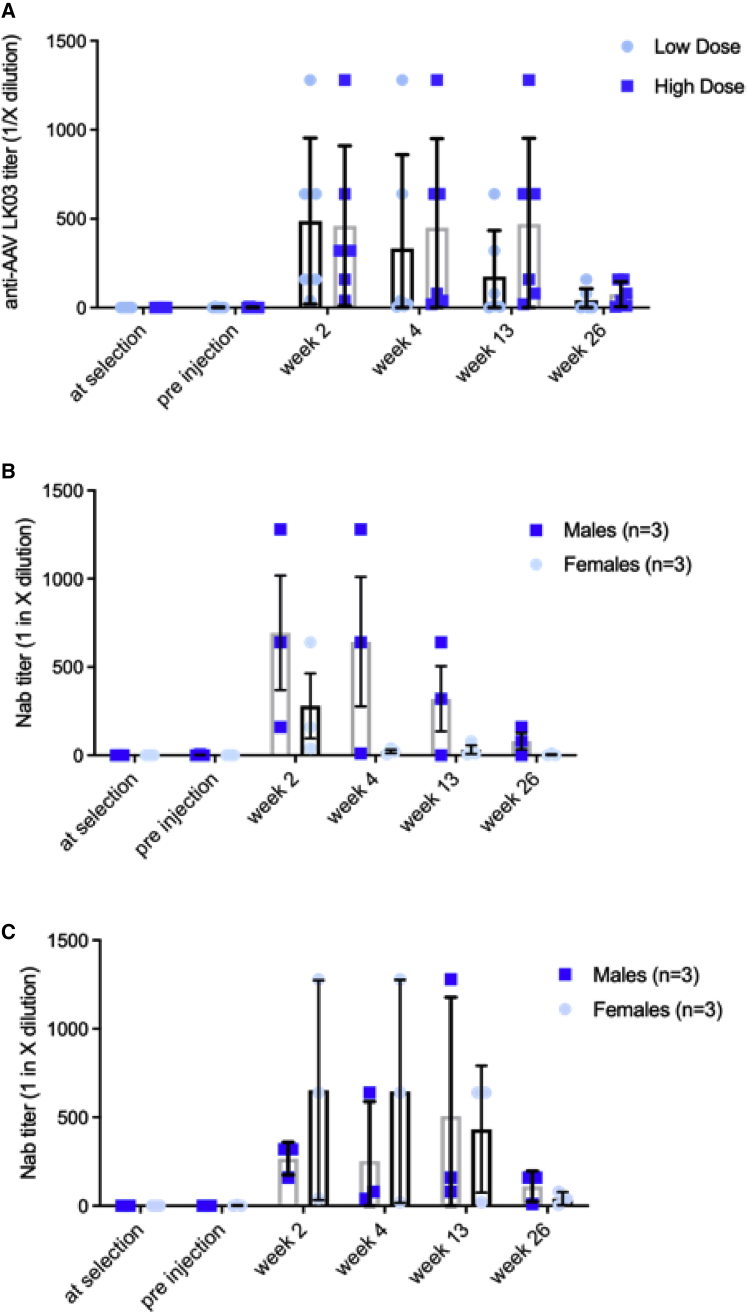
Humoral immune response against AAVLK03 capsid (A) Overall neutralization titers. Gender difference of neutralization titers between low-dose (B) and high-dose (C) groups. Neutralization titers are expressed as 1 in X dilution of serum. Horizontal lines display the mean ± SEM.

In the HD group, the Nab peak plateaued from weeks 2 to 13 before decreasing by week 26. In the LD group, the peak was evident at 2 weeks, followed by a fall in the titers from 4 weeks onward, which was quicker than for the animals in the HD group (Figure 2A). 3/6 animals in the LD group were seronegative at 26 weeks (Figure 2A). Different animals in both LD and HD cohorts mounted variable intensity of humoral immune response (Table S5). There were no marked gender differences in humoral immune responses between the groups at the end of the study (Figures 2B and 2C). In the LD group, females showed a lower production of Nabs compared to males (Figure 2C). The assay at weeks 13 and 26 did not work for an unknown reason for animal 18F and could not be repeated.

#### Cellular immunity

The cellular immune response was assessed prior to vector administration and at weeks 2, 4, 13, and 26 in peripheral blood mononuclear cells (PBMCs). The PBMCs were incubated with 15-mer overlapping peptide libraries for viral protein VP1 of the AAVLK03 capsid and the hOTC protein, and the percentage of helper (CD4^+^) and cytotoxic (CD8^+^) T cells secreting interleukin 2 (IL-2) and interferon gamma (IFN-γ) was detected by flow cytometry. The results showed no cellular response against the viral protein VP1 of the AAVLK03 capsid, suggesting the absence of capsid-specific T cells (Figures S1A−S1C). There was also no response against the hOTC protein. The percentage of T cell-producing cytokines was negligible and very similar to that observed in PBMCs treated with the control Roswell Park Memorial Institute (RPMI) cell culture medium buffer. All PBMC samples at all time points demonstrated capacity to respond to an appropriate stimulus by secreting pro-inflammatory cytokines after incubation with leukocyte activation cocktail (LAC; a polyclonal stimulator) (Figures S1A−S1C).

All samples in all groups yielded results below or near the assay range for IL-1β, IL-2, IL-4, IL-5, IL-6, tumor necrosis factor α (TNF-α), granulocyte colony-stimulating factor (G-CSF), and IFN-γ concentrations with the exception of IL-8. IL-8 concentrations were seen to be variable across all time points and groups, including controls. IL-8 concentrations were lower with the lower doses of AAVLK03.hOTC compared to the controls (Figures S2A−S2C).

### Vector DNA shedding

**Figure 3:**
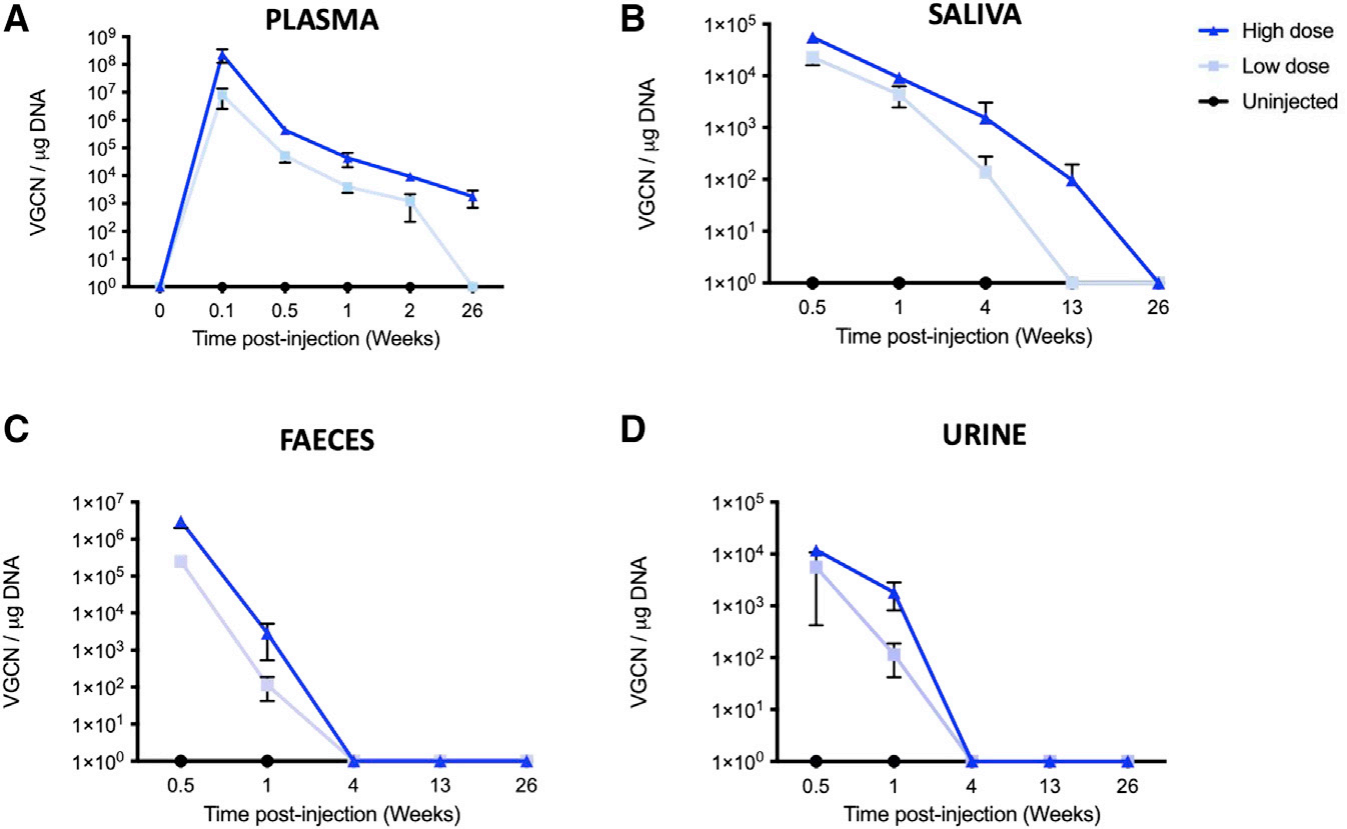
Vector shedding profile of AAVLK03.hOTC Vector shedding profile of AAVLK03.hOTC in (A) plasma, (B) saliva, (C) feces, and (D) urine. Horizontal lines display the mean ± SEM. VGCN, vector genome copy number.

The presence of vector DNA in saliva, urine, and feces was assessed at days 4 and 8 and weeks 4, 13, and 26 and in plasma at baseline; days 1, 4, 8, and 15; and week 26 after AAVLK03.hOTC administration. In plasma samples, the AAVLK03.hOTC vector was quantifiable from day 1 up to day 15 from the LD cohort and from day 1 to week 26 in samples from the HD cohort, with a peak at day 1 (Figure 3A). In the LD group, vector DNA was quantifiable in the plasma from day 1 up to day 15. vg copies were at least 1 log lower in the LD cohort compared to the HD cohort at all time points (Figure 3A). In saliva, the AAVLK03.hOTC viral vector was quantifiable in all swab samples from the LD and HD groups from day 4 and day 8 (Figure 3B). In the LD group, viral vector was detected up to week 4 (only one positive animal), and in the HD group, viral vector was detected up to week 13 (only one positive animal). No vector copies were detected in saliva samples at week 26. In feces samples, the AAVLK03.hOTC vector was detected in LD up to 4 weeks and in HD up to 13 weeks. In urine samples, the vector was detected up to day 8 for the LD cohort and up to week 4 for the HD cohort (Figures 3C and 3D). No vector copies were detected in urine and feces samples from week 13 and week 26.

### Biodistribution

**Figure 4:**
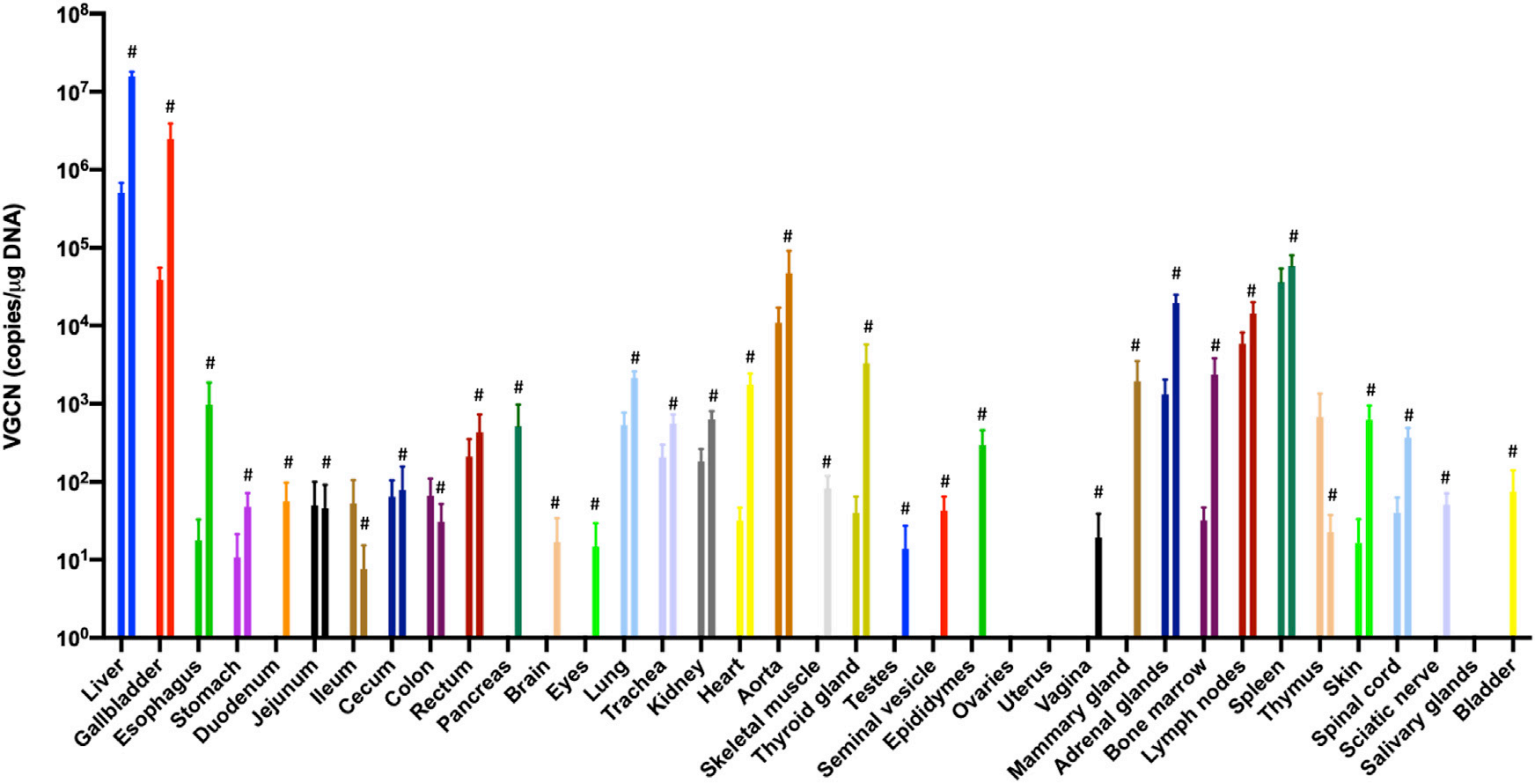
Biodistribution of the AAVLK03.hOTC vector at 26 weeks following single peripheral vein injection For each organ, the low dose and the high dose are represented by the left and right bars, respectively. Horizontal lines display the mean ± SEM. #Bars representing data from the high-dose group.

Vector biodistribution analysis was performed by qPCR on all tissues listed in Table 1, collected at sacrifice 26 weeks after vector administration (Figure 4). The highest number of genome copies (GCs) was found in the liver with an average of 1.6 × 10^7^ GC/μg DNA in the HD group. In contrast, the average GC per microgram in the liver was 31-fold lower in the LD group at an average of 5 × 10^5^ GC/μg DNA. Not surprisingly, gallbladder biodistribution was also high. Most other organs displayed a GC number <1 × 10^3^ GC/μg DNA (Figure 4) except for the spleen and aorta (circa 10^5^ GC/μg DNA), lymph nodes, and adrenals (circa 10^4^ GC/μg DNA). GC number in the central nervous system and genitals was particularly low (undetectable for most samples and <1 × 10^2^ GC/μg DNA for all samples). No vector copies were detected in samples from the control group. No gender difference was observed.

### Supraphysiological liver OTC enzyme activity was sustained in all AAVLK03.hOTC-treated animals

**Figure 5:**
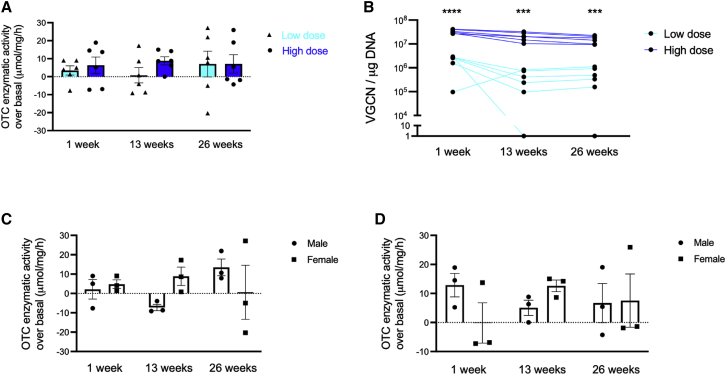
Longitudinal assessment of liver OTC activity (A) Increase of OTC activity after AAVLK03.hOTC injection over physiological OTC activity. (B) VGCN over time. Increase of OTC activity after AAVLK03.hOTC injection over physiological expression according to gender in (C) low- and (D) high-dose groups. The physiological OTC activity is the average of 3 control non-human primates from the same gender. Horizontal lines display the mean ± SEM. (B) Unpaired two-tailed Student’s t test, ∗∗∗p < 0.001; ∗∗∗∗p < 0.0001.

We sequentially measured liver OTC enzyme activity in biopsy samples obtained at 1 and 13 weeks and then at necropsy at 6 months post-administration of AAVLK03.hOTC in treated and control animals. Increased OTC activity was observed in the livers from all treated animals at all time points. In addition, a trend for dose response was observed at 1 and 13 weeks. OTC activity was 2 and 11 times higher in the HD compared with the LD group at 1 and 13 weeks post-administration, respectively. The increase of the OTC activity was similar in both treatment groups at week 26 (Figure 5A; Table S6). GC was almost 1 log lower in the LD group compared to the HD group at all time points (Figure 5B), which was consistent with the log lower dose administered in LD versus HD groups. No gender difference was observed in liver OTC activity either at LD (Figure 5C) or at HD (Figure 5D).

## Discussion

In this good laboratory practice-compliant study, we investigated the safety of AAVLK03.hOTC gene transfer administered intravenously to cynomolgus macaques at doses intended for the first-in-human clinical trial. To our knowledge, this is the first detailed report of a safety study using a vector with an engineered human liver-tropic AAV capsid in non-human primates. There were no adverse events or abnormal histological lesions observed at the doses intended for clinical use, supporting safety of this vector in juvenile animals. It is expected that as for wild-type AAV capsids, the testing and dose extrapolation of engineered capsids in animal models for human use will be reliable.^7^

The biodistribution of AAVLK03.hOTC confirmed a remarkable liver tropism with partial biodistribution to the spleen. There was no transduction of the central nervous system and no or very low transduction of the genital organs. AAV8 has been historically the first serotype demonstrating long-lasting efficacy of AAV liver-directed gene therapy.^9^^,^^10^ AAVLK03 was shown to selectively transduce primary human hepatocytes *in vivo*, which was 12-fold better than AAV8 in chimeric mouse-human livers.^15^ However, AAVLK03 was not found to be superior to AAV8 in non-human primates s in terms of liver tropism.^24^

The AAVLK03.hOTC vector was quantifiable from day 1 up to day 15 from the LD cohort and from day 1 to week 26 in plasma samples from the HD cohort, with a peak at day 1 (Figure 3A). In comparison, peripheral vein administration of the (2 × 10^12^ vg/kg) resulted in an average of 3.6 ± 0.9 × 10^9^ vg/mL of self-complementary (sc)AAV2/8-LP1-hFIXco DNA in the plasma on day 1 following vector administration, which gradually declined to undetectable levels by day 10 in non-human primates.^25^ In a preclinical study assessing a HD of 5 × 10^13^ vg/kg of AAV5 vector against acute intermittent porphyria in macaques, the maximal vector DNA concentrations in plasma were observed at 8 h after vector injection and with a gradual decrease observed up to 30 days. In our study, vg copy numbers (VGCNs) in all body fluids assessed were more than 1 log higher in the HD cohort of AAVLK03 compared with VGCNs observed with 5 × 10^13^ vg/kg of the AAV5 vector.^26^ Lower VGCNs were detected in urine, feces, and saliva samples in all studies; however, VGCNs detected in saliva, stool, and urine with AAVLK03 vector are at least 1 log higher than VGCNs observed with an AAV8 vector at the same dose (2 × 10^12^ vg/kg).^25^

Biodistribution of AAVLK03.hOTC showed the highest number of VGCNs in the liver and the gallbladder as expected, followed by spleen, aorta, lymph nodes, and adrenals. In a preclinical study using an AAV8 vector at a dose of 2 × 10^12^ vg/kg, liver was also the main transduced organ followed by adrenals, spleen, aorta, and heart. Similarly to our observation with AAVLK03.hOTC, transduction levels were lowest in the central nervous system and the genital organs using AAV8.^25^

Immune responses to AAV can be classified as innate and adaptive. Adaptive immunity entails humoral and cell-mediated reactions. Several studies have shown the seroprevalence of Nabs directed against various AAV serotypes among the general population.^27^^,^^28^ This can be caused by multiple infections from various wild-type AAV serotypes.^27^^,^^29^^,^^30^ Another explanation can be the wide cross-reactivity of Nabs between AAV serotypes caused by homology of the amino acid sequence.^28^ In this study, after single administration of AAVLK03.hOTC, the humoral immune response had a maximal titer at week 2 post-administration and was similar in both genders.. These titers decreased rapidly over the 26-week observation period with undetectable titers in some animals and a significant decrease in most animals especially in the LD group. The transient and limited immune response in our study supports the belief that engineered AAV capsids can evade Nabs more easily or generate Nabs, which rapidly decrease overtime.^15^^,^^16^ This might explain why anti-AAVLK03 Nabs are particularly low in childhood.^19^

AAV vectors can also trigger the cellular immune response.^31^ Immune-mediated destruction of transduced hepatocytes is associated with CD8^+^ T cell responses to AAV capsid antigens, which are processed and presented by major histocompatibility complex (MHC) class I.^32^ AAV vectors with a single-stranded (ss)DNA genome are able to stimulate the innate immune system through the Toll-like receptor (TLR)9/myeloid differentiation primary response gene 88 (MyD88) and type I IFN cascades.^33^ However, only a mild and highly transient immune response has been observed for ssAAVs regardless of capsid sequence, whereas changing the genome to scAAV increases innate immunity in a TLR9-dependent manner.^34^ In our study, the single administration of AAVLK03.hOTC did not show any cell-mediated immune response against either AAV capsid or OTC protein from isolated PBMCs. Similarly, a T cell response was not detected in non-human primates transduced with 2 × 10^12^ vg/kg of AAV8 in a hemophilia B study.^25^ However, increased AAV8 capsid-reactive T cells were seen within 3 months after the delivery in a human clinical trial in patients injected with both 6 × 10^11^ and 2 × 10^12^ vg/kg doses.^9^ Long-term monitoring showed capsid-reactive T cells detected in peripheral blood for the first year following injection.^10^ Thus, a cellular immune response may be present in human trials despite an absence of its detection in preclinical studies.

Asymptomatic elevation in liver function tests is one of the adverse events associated with liver-targeted AAV gene therapy. Recombinant AAV encoding the hFIX gene caused transient elevations in transaminases in 2 out of 5 non-human primates in another preclinical study.^35^ In contrast, in a long-term safety and efficacy study for hemophilia B, macaques injected with 2 × 10^12^ vg/kg of AAV8 vector had normal liver function tests for the 5 years of follow-up.^25^ However, the subsequent clinical trial revealed an asymptomatic transient increase of transaminases between week 7 and week 10 in 4 out of 6 patients injected with the same vector at the same dose.^10^ A phase I/II clinical trial in hemophilia A with AAVLK03 also revealed that three participants had elevation of transaminases.^36^ Therefore, despite the absence of increased transaminases in our preclinical study, careful monitoring is still warranted in future human clinical trials.

It has been previously shown that small differences in OTC activity can be responsible for the variability of the clinical phenotype in humans, which ranges between mild and severe.^37^, ^38^, ^39^, ^40^ This study was performed in juvenile animals mimicking the growth of a pediatric liver. Interestingly, the increase in OTC activity following vector administration was sustained throughout the 26-week study period with a trend for dose response at early time points. This trend was surprisingly not observed at the end of the study, although there was larger variability in values at this time point compared to others, affecting all experimental groups including controls, which might have masked this effect. A sampling effect and the uneven OTC expression throughout the liver due to metabolic zonation could partially explain this variability. This contrasts with a significant dose response of VGCN at all time points. The GC levels in the growing liver of juvenile macaques also remained high over the 26 weeks with only a mild decline as expected, suggesting only a partial loss of episomal transgene copies in the growing liver. Cynomolgus macaques can live as long as 40 years (average 27 years).^41^ Female and male cynomolgus macaques reach sexual maturity at 4 and 7 years old, respectively.^42^ The animals used in this study were 14 to 18 months at the time of injection and considered as juvenile animals with ongoing liver growth, as seen in young pediatric patients. Altogether, these findings support the potential benefit of AAVLK03.hOTC in pediatric OTCD patients.

Overall, this study of intravenous AAVLK03.hOTC in juvenile cynomolgus macaques demonstrated safety of the tested vector with no toxicity, an excellent liver tropism with limited off-target biodistribution, a limited humoral immune response, and sustained increase in the enzyme activity in vector-treated animals. This study supports the clinical development of AAVlk03.hOTC for a first-in-human clinical trial.

## Materials and methods

### Vector production and study design

The codon-optimized hOTC (hco*OTC*) transgene was generated by GenScript (Piscataway, NJ, USA) using the OptimumGene algorithm. The *hcoOTC* sequence is available as Supplemental information.

AAV (AAV-LK03) with the OTC transgene was produced by the Clinical Vector Core at the Children’s Hospital of Philadelphia. The process and materials were identical to those used in good manufacturing practice (GMP) manufacturing following Food and Drug Administration recommendations that materials designated for pharmacology/toxicology safety studies are GMP-process comparable. Briefly, the AAVLK03.hOTC vector was generated by triple calcium phosphate transient transfection of adherent human embryonic kidney (HEK) epithelial cells (HEK293) expanded in tissue-culture flasks and roller bottles prior to transfection. Cells were transfected using three plasmids that included the transgene plasmid (pAAV2-LPhOTC), Adenoviral-helper plasmid (pCCVC-AD2HPv2), and capsid plasmid (LK03). Post-transfection, the media were replaced with serum-free medium, and at the optimal time point post-transfection, cells and media were harvested. Crude harvest, including cells and media, was concentrated by hollow fiber tangential flow filtration (TFF), and cells were lysed by microfluidization (MF), treated with Benzonase, and clarified by 0.2 μ filtration. AAV vector in the post-MF-filtered material was purified using ion exchange chromatography. Transgenes containing particles were fully separated from empty particles using cesium-chloride gradient centrifugation and collected. Viral vector derived from multiple batches was pooled, diafiltered into final formulation buffer (180 mM NaCl, 10 mM Na phosphate, 0.001% poloxamer 188) by TFF, sterile 0.2 μ filtered, aseptically filled in 1.5 mL cryovials at 1.0 and 0.3 mL per vial, and stored at −80°C pending completion of QC testing and quality analysis (QA) release.

The AAVLK03.hOTC was administered via a single intravenous injection. The administration was performed in the left cephalic vein in both LD and HD groups and in the left saphenous vein in controls. After the injection, 1 mL of diluent provided by the manufacturer was flushed through the catheter. The amount of the test item to be administered was calculated according to the body weight recorded on the administration day. Control animals were administered with diluent following the same administration pattern as that followed for animals treated with the test item. Administration volume was set as 0.8 mL/kg (PBS), 0.36 mL/kg (2 × 10^12^ vg/kg), and 0.8 mL/kg (2 × 10^13^ vg/kg) in controls and LD and HD groups, retrospectively. Duration of monitoring post-injection was 26 weeks.

### Animal procedures

All animal procedures were approved by the Ethical Institutional Committee at Envigo and were compliant with Animal Research:Reporting of In Vivo Experiments (ARRIVE) guidelines. Naive cynomolgus monkeys (*Macaca fascicularis*; supplier, KHI Bioservices, Hong Kong; breeder, Nafovanny, Vietnam) were pre-screened to confirm absence of Nabs. The females were nulliparous and nonpregnant. 10 males and 10 females (9 males and 9 females were allocated to treatment groups) were between the age of 14 and 18 months at the beginning of treatment. In the interim period, their body-weight ranges were 1.5−2.2 kg and 1.6−2.3 kg in males and females, respectively. Animals were seronegative against AAV-LK03 and were allocated at random to the two treatment groups.

### Safety and toxicity assessments

#### In-life analysis

The observations listed below were recorded. Viability/mortality/cage-side observations were done twice a day. Detailed clinical signs, including revision of the injection area, were recorded on the day of administration and weekly after dosing. Food consumption was evaluated by visual assessment and described as none, normal, and low. Daily check was done from the pre-test period to 26 weeks following the day of administration. Body weight was assessed weekly from pre-test, for 26 weeks after administration, and before sacrifice (fasted).

Signs are reported in four categories: detailed clinical signs, veterinary inspection, dose-site observations, and pen-side observations. Clinical observations are presented for all animals showing signs, providing detail of type of sign, day or week of occurrence, and information on the duration of the sign.

All animals had an indirect ophthalmoscopic evaluation (Welch Allyn; model number [no.] 12500) at the pre-test period and in week 26. Observation areas were cornea, lens, conjunctiva, sclera, iris, and fundus.

All animals had electrocardiograms at the pre-test period and in week 26. Electrocardiograms (single snapshots) were obtained using Einthoven (I, II, and III) and Goldberger (aVR, aVL, and aVF) leads. The heart rate, P-wave duration and amplitude, PQ interval, QRS interval, and QT interval were measured using a representative section of the electrocardiogram from lead II. Correction of the QT interval for heart rate (Fridericia) was also calculated.

#### Clinical laboratory investigations

The samples were collected early in the working day to reduce biological variation caused by circadian rhythms. The monkeys were fasted for blood collections. Blood samples were drawn from the femoral vein. Blood and urine samples were collected at the pre-test period and weeks 3−4, 12−13, and 25−26. The assay was performed at Envigo CRS, S.A.U., under internal laboratory QC conditions to assure reliable test results.

*Hematology.* Samples were collected in 0.5 mL tri-potassium-EDTA tubes. The following parameters were determined using an ADVIA 120 hematology analyzer: erythrocyte count (RBC), hematocrit (Hct), hemoglobin (Hb), mean corpuscular Hb (MCH), mean corpuscular Hb concentration (MCHC), mean corpuscular volume (MCV), platelet (thrombocyte) count (Plt), reticulocyte count (absolute and relative; Ret), total leukocyte count (WBC), differential leukocyte count (neutrophils [N], lymphocytes [L], monocytes [M], eosinophils [E], and basophils [B]), and large unstained cells (LUCs).
*Coagulation.* Samples were collected in 0.5 mL 3.2% sodium citrate (1 part anticoagulant to 9 parts blood) tubes in order to obtain the plasma. The following parameters were determined: prothrombin time (SPT) and activated partial thromboplastin time (SAPT).
*Clinical biochemistry.* Samples were collected in 0.8 mL lithium heparin tubes in order to obtain the plasma. The resultant plasma was analyzed using the Cobas 6000 analyzer for the following parameters: alanine aminotransferase (ALT); creatinine (Creat); alkaline phosphatase (ALP); albumin (Alb); Alb/globulin ratio (A/G); aspartate ALT (AST); bilirubin, total (Bili); calcium (Ca); chloride (Cl); cholesterol, total (Chol); creatine kinase (CK); gamma-glutamyltransferase (gGT); globulin∗; glucose (Gluc); phosphorus, inorganic (Phos); potassium (K); protein, total (Total Prot); sodium (Na); triglycerides (Trig); and urea.
*Urine analysis.* The following parameters were determined: relative gravity (SG1), color (COL), appearance (App), pH, nitrite (NITE), protein (PROT), Gluc, ketone (Keto), urobilinogen (Urob), Bili, erythrocytes (UBld), and leukocytes (UWBC).

### Efficacy assessments

OTC enzyme activity was measured by a colorimetric assay, which detects the formation of L-citrulline. Liver tissue was prepared as a 1% (w/v) homogenate in deionized water, and fetal liver pellet was prepared as a 3%−5% (w/v) suspension in deionized water using a tissue homogenizer. Samples were stored at −70°C until the assay. Citrulline is produced by the OTC reaction, similar to the reaction *in vivo*, and was quantitated using a phenazone diacetyl reagent producing a strong yellow color read on a spectrophotometer set at 464 nm. CPS was measured as a control enzyme.

OTC + ornithine
Carbamoyl phosphate → citrulline
Citrulline + phenazone/diacetyl reagent → yellow product (absorbance [Abs.] 464 nm)

600 μL of working phenazone diacetyl reagent was added to 100 μL of the assay supernatants in 5 ml stoppered glass tubes and for the standards. They were heated at 100°C for 10 min in the dark. Then they were cooled in ice water and then allowed to come to room temperature before reading. The color was stable at room temperature. The absorbance was measured at 464 nm using a glass microcuvette. Then the cuvette was rinsed with phenazone diacetyl reagent and the sensitivity of the spectrophotometer adjusted using the standard containing no citrulline. Results were expressed in micromoles of citrulline/hour/milligram of proteins.

### Viral shedding and biodistribution

DNA was extracted from tissues issuing the QIAamp Fast DNA Tissue Kit (ref. 51404; QIAGEN). Assays results were reported by Real-time PCR Equipment Mx3005P Stratagene (Agilent Technologies). For tissues, results were adjusted to double-stranded vector copies/microgram genomic DNA. Those tissue samples analyzed with <500 ng genomic DNA/reaction are noted in Results. For plasma, urine, feces, and saliva (shedding samples), results were adjusted to ss vector copies. Results are expressed considering the volume of sample (copies/100 μL plasma or urine, copies/100 mg feces, and copies/saliva swab).

### Necropsy

Animals were euthanized with intravenous pentobarbital overdose and necropsied and tissues harvested for comprehensive histopathologic examination. Table 1 lists tissues that were collected and examined histologically. Organ weights were presented both as absolute/unadjusted and adjusted for terminal body weight using the weight recorded on the day of necropsy.

#### Histology

All organ and tissue samples to be examined by the study pathologist were fixed and sent to ProPath for processing. The samples were processed, embedded, cut at a nominal thickness of 4−5 μm, and stained with hematoxylin and eosin. After evaluation, any remaining tissues and organs were stored for archiving. The bone marrow smears were stained using the May-Grunwald Giemsa method and stored for possible further investigation.

#### Pathology

Slides of all organs and tissues collected at necropsies of all animals were examined by a veterinary pathologist at Envigo CRS, S.A.U. Where possible, the microscopic findings were correlated with the gross observations. A peer review of microscopic findings was performed in accordance with Envigo standard operating procedures.

### Immunological assessments

#### Humoral

Blood samples (nominally, 500 μL) were collected from the femoral vein from all animals on the following occasions: pretreatment and weeks 2, 4, 13, and 25−26. The blood samples were maintained at room temperature until centrifugation. Each blood sample was centrifuged at 1,500 × *g* during 15 min at room temperature (11 to 68 min from collection) after coagulation in order to obtain around 200 μL of serum. The resulting serum was transferred to a polypropylene vial and then was immediately frozen in dry ice and stored below −80°C ± 10°C until dispatch.

Human hepatocellular carcinoma HuH-7 cells were seeded at 5 × 10^4^ cells per well on a 48-well plate and left for at least 7 h before transduction. Before use, sera were heat inactivated at 56°C for 30 min. Precipitate resulting from heat inactivation was removed by centrifugation at 15,000 × *g* for 10 min. On the day of transduction, dilution of serum sample was performed in Dulbecco’s modified Eagle’s medium (DMEM) (Gibco, Invitrogen, Grand Island, NY, USA) without fetal calf serum (FCS) (JRH Biosciences, Lenexa, KS, USA) starting from 1 in 5 and then in 2-fold serial dilutions to 1:640. The diluted serum samples were incubated for 1 h at 37°C with AAVLK03.hAAT.GFP diluted in an equal volume of DMEM. AAVLK03 was incubated at the same concentration to reach a predetermined final multiplicity of infection (MOI) into a 100-μL final volume for transduction. The optimal transduction for the assay was reached at an MOI of 1,000 vg/cell for AAVLK03. Purified immunoglobulins (Octagam 10% [v/v]; Octapharma, Manchester, UK) were used as positive control. Each assay was run in duplicate. The next day, wells were complemented with DMEM and 10% (v/v) FCS. Fluorescence-activated cell sorting BD FACSVerse (BD Biosciences, San Jose, CA, USA) analysis of the GFP signal was performed 72 h post-transduction. Samples were considered positive when a 1:5 dilution of serum reduced the vector transduction by 50% or more. The Nab titer was determined as the highest positive serum dilution.

#### Cellular

Peripheral blood T cell responses against hOTC and the AAVLK03 capsid were performed by the BD FACSCanto II Flow Cytometer with FACSDiva software (BD Biosciences), according to previously published methods, using peptide libraries specific for AAVLK03 capsid and the hOTC transgene. Percentages of cells were reported to two decimal places. Frequencies of helper (CD4^+^) and cytotoxic (CD8^+^) T cells producing cytokines IL-2 and IFN-γ^+^ in PBMCs treated with RPMI cell culture medium (non-stimulated), LAC (polyclonal positive control), peptide pool from the capsid, and peptide pool from the transgene were calculated using the appropriate template of FCS Express version 6.

#### Cytokine determination

Totally, 90 blood samples (nominally, 250 μL) were collected in tubes with EDTA K_2_ from the femoral vein from all animals on the following occasions: day 1 (predose, 4, 8, and 24 h after administration) and weeks 25−26. The blood samples were maintained at room temperature until centrifugation. Each blood sample was centrifuged at 2,000 × *g* during 10 min at 4°C in order to obtain plasma. The resulting plasma was transferred to a polypropylene vial and then was immediately frozen on dry ice and stored below −80°C ± 10°C until dispatch. Plasma levels of IL-1β, IL-2, IL-4, IL-5, IL-6, IL-8, TNF-α, G-CSF, and IFN-γ were analyzed at Envigo CRS (UK). This assay is based on the Luminex xMAP technology. Data are presented as concentrations in whole matrix expressed in picograms per milliliter.

### Statistical analysis

Statistical analysis has been performed; however, due to the small sample size, there may not be adequate power to identify biologically relevant differences as being statistically significant. All statistical analyses were carried out separately for males and females. For all parameters, the analyses were carried out using the individual animal as the basic experimental unit. The following data types were analyzed at each time point separately: body weight, electrocardiography, hematology, blood chemistry, urine analysis, macropathology, and organ weights (absolute and adjusted for terminal body weight). Comparison of continuous variables between two experimental groups was performed using the Student’s two-tailed t test. p values < 0.05 were considered statistically significant. Standard error of the mean (SEM) was used as the indicator of dispersion.

## Supplemental information

Document S1. Figures S1 and S2, Tables S1–S6, and Supplemental information

Document S2. Article plus supplemental information
