# Supplementary material for: Safety and efficacy of an engineered hepatotropic AAV gene therapy for ornithine transcarbamylase deficiency in cynomolgus monkeys
Source: Mol Ther Methods Clin Dev. 2021 Sep 14;23:135–46. doi: 10.1016/j.omtm.2021.09.005 (PMC8517016; doi:10.1016/j.omtm.2021.09.005)
Supplement: Document S1. Figures S1 and S2, Tables S1–S6, and Supplemental information [file mmc1.pdf]

OMTM, Volume *23*

Supplemental information

Safety and efficacy of an engineered hepatotropic AAV gene therapy for ornithine transcarbamylase deficiency in cynomolgus monkeys

Julien Baruteau, Sharon C. Cunningham, Berna Seker Yilmaz, Dany P. Perocheau, Simon Eaglestone, Derek Burke, Adrian J. Thrasher, Simon N. Waddington, Leszek Lisowski, Ian E. Alexander, and Paul Gissen

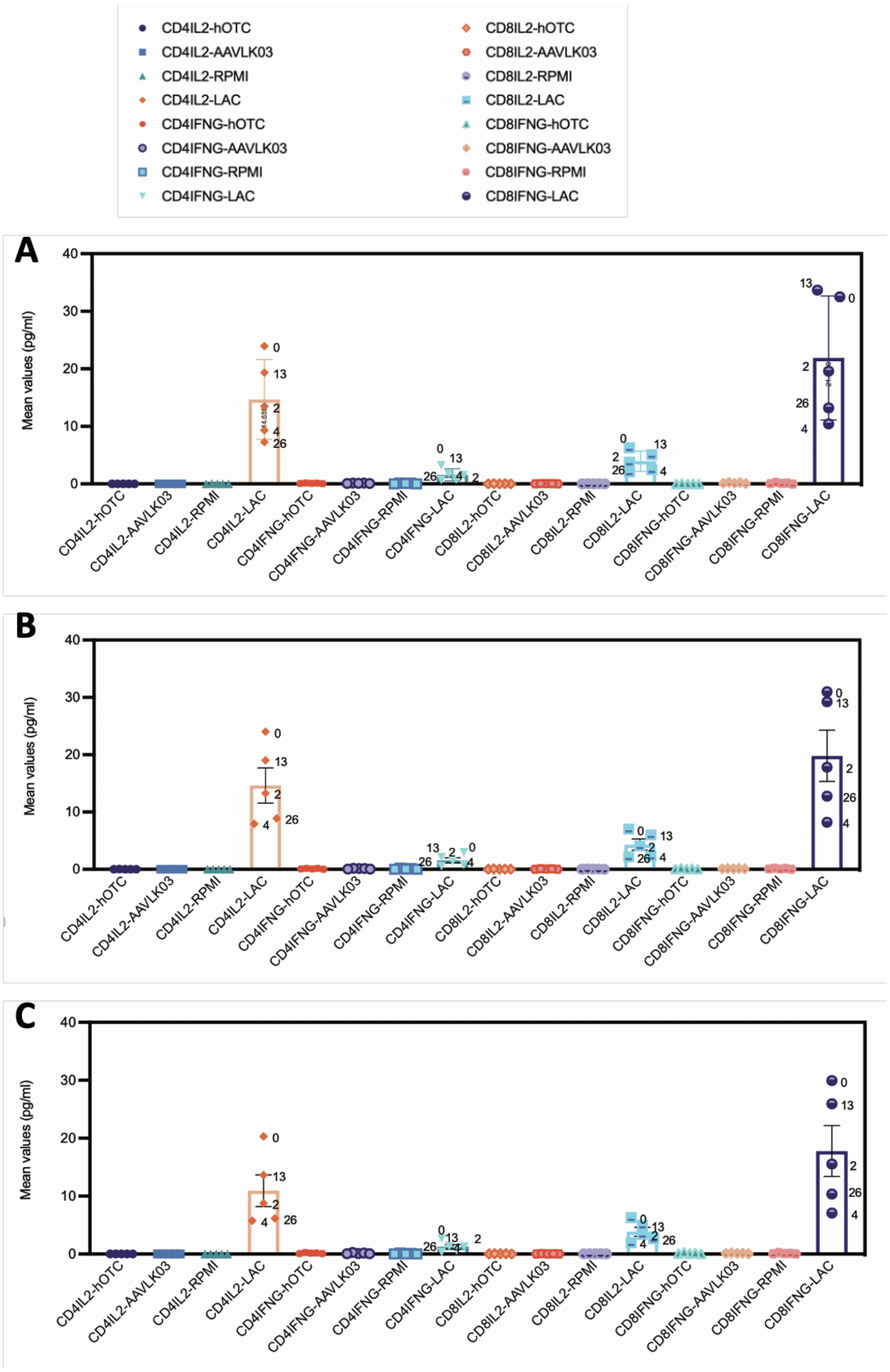

Supplementary Figure 1. T Cell Responses in PBMCs Treated in Vitro with the Pool of Peptides AAVLK03, hOTC, RPMI and LAC in A) control group B) low dose group C) high dose group. Horizontal lines display the mean ± SEM. Timepoints are mentioned as 0,2,4,13 and 26 weeks for the ones different from zero. hOTC: human OTC protein; CD4: Helper T Cells; CD8: Cytotoxic T cells; IL2: Interleukin 2; IFNG: Interferon gamma; PBMC: Peripheral blood mononuclear cells; RPMI: Roswell Park Memorial Institute cell culture medium ; LAC: Leukocyte Activation Cocktail.

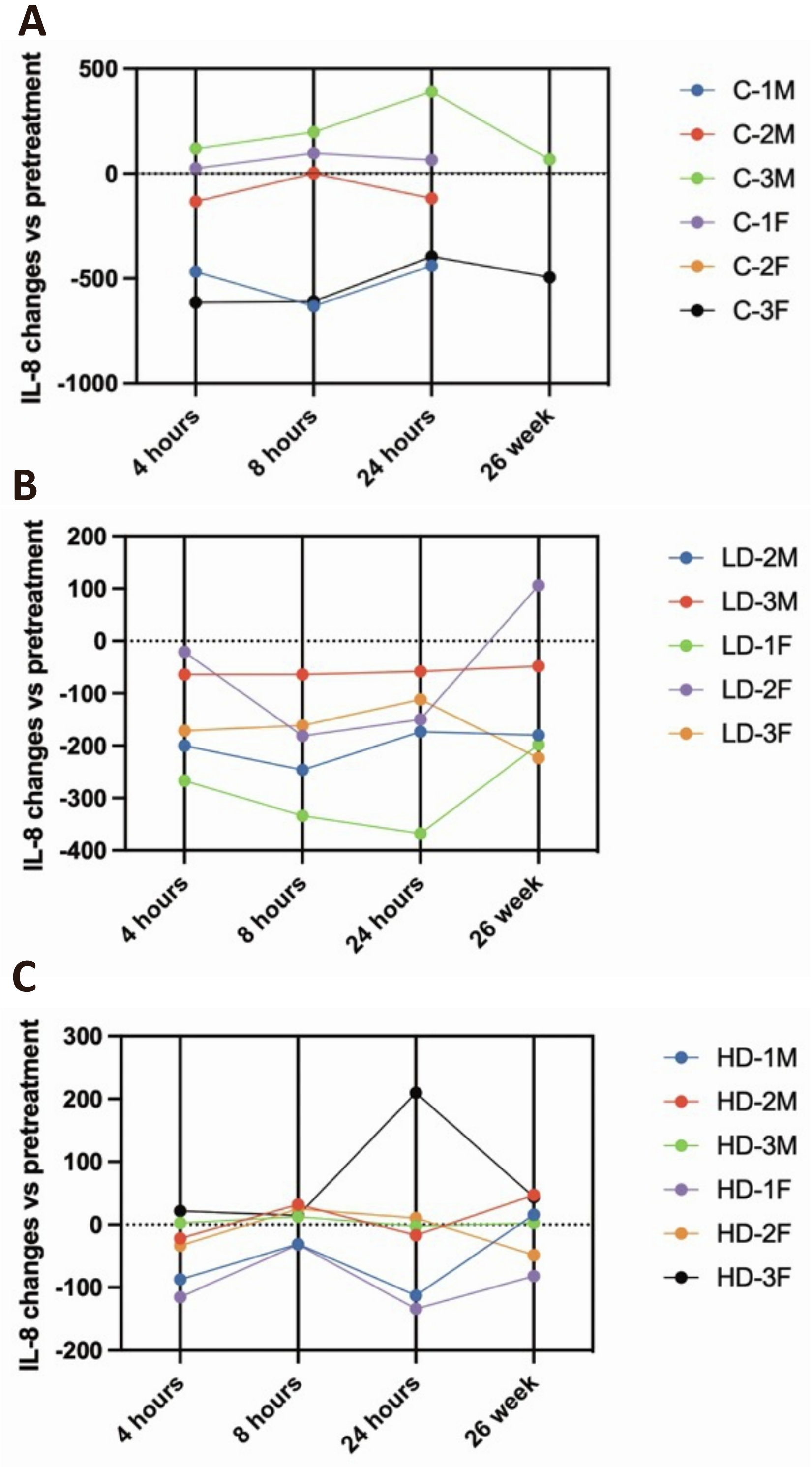

Supplementary Figure 2. Change in the IL-8 levels at 4 hours, 8 hours, 24 hours and 26 weeks after vector injection versus pretreatment levels in A) control group B) low dose group C) high dose group. Each line represents the changes of each animal at the mentioned time points. IL-8: Interleukin 8; C: Control group; LD: Low dose group; High dose group; M: Male; F: Female.

Supplementary Table 1. Clinical laboratory parameters at baseline

C: Control; LD: Low dose; HD: High dose; M:Male; F: Female; Hb: Haemoglobin; Hct: Haematocrit; Ret: Reticulocyte; sPT: Prothrombin time; sAPT: Activated partial thromboplastin time; Cre: Creatinine; Na: Sodium; K: Potassium

	Hb (g/dL)	Hct (L/L)	Ret (%)	WBC (x109/L)	Plt (x109/L)	sPT (secs)	sAPT (secs)	ALT (U/L)	AST (U/L)	CK (U/L)	Urea (mmol/L)	Cre (µmol/L)	Na (mmol/L)	K (mmol/L)	Albumin (g/L)	Globulin (g/L)
**C-1M**	11.9	0.427	2.18	17.22	652	11.3	25.8	32	49	126	5.4	40	151	3.8	43	26
**C-2M**	13.7	0.482	1.14	17.14	478	11.6	25.7	31	51	201	8.21	42	149	4.2	44	28
**C-3M**	13.2	0.483	1.16	12.27	473	11.2	24.9	29	85	210	7.56	48	148	2.8	47	31
**C-1F**	13	0.439	0.7	16.24	384	12.2	25.5	43	42	193	7.93	39	147	3.8	49	27
**C-2F**	12.7	0.419	0.6	15.54	414	11.6	25.5	31	43	119	6.82	46	147	3.7	48	27
**C-3F**	13	0.416	0.76	9.33	452	12.7	24.8	40	45	107	9.02	47	147	3.6	48	27
**LD-1M**	13.6	0.485	1.32	15.41	504	11.2	24.7	37	60	187	10.36	55	144	3.6	44	34
**LD-2M**	12	0.408	1.59	8.19	445	11.6	23.8	37	35	83	10.69	52	144	3.4	44	26
**LD-3M**	13	0.438	1.06	11.06	673	11.3	23.2	35	32	134	8.08	38	141	3.8	42	32
**LD-1F**	14.5	0.484	0.91	11.69	486	11.6	26.2	22	29	118	6.84	43	154	4.3	50	29
**LD-2F**	14	0.428	0.55	13.52	307	12.2	29.5	26	37	307	8.81	52	146	3.4	45	27
**LD-3F**	13.5	0.423	0.39	10	366	11.4	27	37	46	110	7.69	41	147	4	45	26
**HD-1M**	12.9	0.443	1.25	13.81	614	12.3	23.3	36	40	179	6.57	55	144	3.4	45	29
**HD-2M**	12.7	0.444	1.24	18.59	505	11.3	27.6	47	47	156	6.01	55	147	3.4	47	30
**HD-3M**	11.8	0.426	1.49	14.69	417	12.6	INS	56	48	115	8.06	47	147	3.5	47	31
**HD-1F**	13.8	0.443	0.78	19.2	408	11.7	23	30	38	166	8.13	51	147	3.7	42	26
**HD-2F**	13	0.425	0.57	14.8	438	11.8	24.1	24	43	119	8.83	58	146	3.8	47	25
**HD-3F**	13.1	0.423	0.42	12.55	293	10.8	22	31	40	182	6.42	56	148	4	46	28

Supplementary Table 2. Clinical laboratory parameters at week 4. C: Control; LD: Low dose; HD: High dose; M:Male; F: Female; Hb: Haemoglobin; Hct: Haematocrit; Ret: Reticulocyte; sPT: Prothrombin time; sAPT: Activated partial thromboplastin time; Cre: Creatinine; Na: Sodium; K: Potassium

	Hb (g/dL)	Hct (L/L)	Ret (%)	WBC (x109/L)	Plt (x109/L)	sPT (secs)	sAPT (secs)	ALT (U/L)	AST (U/L)	CK (U/L)	Urea (mmol/L)	Cre (µmol/L)	Na (mmol/L)	K (mmol/L)	Albumin (g/L)	Globulin (g/L)
**C-1M**	12	0.412	1.57	9.82	581	11	23.5	37	48	128	4.86	39	143	3.8	44	26
**C-2M**	12.4	0.414	0.95	19.07	420	11.4	25.3	35	39	212	3.73	38	145	4	43	28
**C-3M**	12.2	0.415	1.55	11.74	383	10.6	22.6	21	53	138	5.98	39	144	4	40	35
**C-1F**	11	0.371	1.22	22.46	377	12.6	23.2	47	72	260	5.3	35	142	4.3	39	22
**C-2F**	12.2	0.411	1.13	14.41	432	11.4	22.7	31	37	195	4.84	40	147	3.3	45	27
**C-3F**	12.1	0.401	1.47	12.04	500	11.6	22.4	39	35	174	6.8	40	145	3.2	42	29
**LD-1M**	13.5	0.45	0.55	16.29	420	10.4	23.3	31	40	191	5.25	55	141	3.3	40	35
**LD-2M**	11.7	0.387	0.71	10.78	352	10.9	22.1	37	25	135	5.92	49	146	3.8	40	29
**LD-3M**	12.9	0.413	0.88	13.92	503	10.7	21	30	20	134	5.86	40	144	4.1	41	33
**LD-1F**	13.4	0.451	1.26	17.46	459	11.2	23.6	26	27	296	5.22	45	145	4.3	45	28
**LD-2F**	12.7	0.416	1.21	17.32	402	11	25.3	26	28	174	5.84	49	144	3.4	42	31
**LD-3F**	13.1	0.41	0.82	13.84	402	10.8	25.3	33	33	150	5.79	39	147	4.1	41	31
**HD-1M**	12.9	0.422	1.17	11.8	565	11.5	21.9	43	32	209	5.23	52	144	3.4	44	29
**HD-2M**	12.8	0.433	0.85	11.98	490	10.5	24.2	65	40	203	4.57	53	146	4	47	33
**HD-3M**	11.9	0.405	0.96	13.9	310	12	22	41	32	185	5.77	50	146	3.9	45	33
**HD-1F**	12.6	0.416	1.08	14.91	403	10.8	22.6	40	38	206	5.78	47	145	4.6	43	27
**HD-2F**	12.2	0.411	1.05	16.94	457	11	21.6	41	39	211	4.5	63	147	4.3	42	34
**HD-3F**	12.9	0.418	0.92	9.81	348	10.2	20.9	30	37	226	7.84	55	147	4.2	46	30

Supplementary Table 3. Clinical laboratory parameters at week 13. C: Control; LD: Low dose; HD: High dose; M:Male; F: Female; Hb: Haemoglobin; Hct: Haematocrit; Ret: Reticulocyte; sPT: Prothrombin time; sAPT: Activated partial thromboplastin time; Cre: Creatinine; Na: Sodium; K: Potassium

	Hb (g/dL)	Hct (L/L)	Ret (%)	WBC (x109/L)	Plt (x109/L)	sPT (secs)	sAPT (secs)	ALT (U/L)	AST (U/L)	CK (U/L)	Urea (mmol/L)	Cre (µmol/L)	Na (mmol/L)	K (mmol/L)	Albumin (g/L)	Globulin (g/L)
**C-1M**	12.1	0.402	1.09	15.67	557	11.6	25.6	25	46	66	6.44	37	144	3.8	42	29
**C-2M**	13.4	0.437	0.64	20.88	442	11.3	25.9	27	35	80	7.66	37	145	3.8	44	28
**C-3M**	13.3	0.451	0.63	16	410	10.8	24.1	18	52	69	5.81	39	140	3.9	41	30
**C-1F**	12.9	0.451	0.58	20	370	12.7	24	31	32	110	6.81	37	147	4.5	46	25
**C-2F**	12.4	0.419	0.58	14.41	481	12.2	26.5	30	38	90	2.62	49	146	3.4	46	25
**C-3F**	12.3	0.409	0.75	13	545	12.3	23.9	30	34	109	7.18	51	142	3.6	43	27
**LD-1M**	13.4	0.452	0.53	17.31	527	10.7	24.8	17	40	77	6.61	57	142	4	33	31
**LD-2M**	12.3	0.398	0.6	10.76	409	11	24.5	26	27	75	5.08	49	143	4.2	42	26
**LD-3M**	13.7	0.429	0.43	14.28	470	11.1	23.6	26	24	178	4.05	42	141	3.4	42	29
**LD-1F**	13.8	0.458	0.65	11.15	470	11.3	26	19	31	135	6.37	53	145	3.9	46	25
**LD-2F**	13.2	0.429	0.47	13.51	345	11.8	27.3	21	30	92	8.56	54	144	3.6	45	22
**LD-3F**	12.8	0.406	0.38	16.45	378	10.8	27.2	26	39	120	7.42	44	143	4.2	43	25
**HD-1M**	12.88	0.419	0.53	10.94	531	11.7	23.4	32	33	133	6.97	57	144	3.3	46	24
**HD-2M**	12.8	0.419	0.49	11.2	423	11	27	35	37	88	6.64	61	145	3.4	47	31
**HD-3M**	12.4	0.406	0.45	10.12	311	12.1	23.2	38	31	94	6.37	51	142	3.9	46	29
**HD-1F**	13.5	0.452	0.67	19.04	391	11.7	23.7	27	34	112	7.49	52	147	3.8	45	25
**HD-2F**	12.6	0.413	0.46	18.78	437	10.9	23	31	34	85	5.68	60	144	4.1	48	21
**HD-3F**	12.9	0.427	0.55	10.34	289	11.2	23.7	28	38	75	8.8	64	145	4.1	52	23

Supplementary Table 4. Clinical laboratory parameters at week 26. C: Control; LD: Low dose; HD: High dose; M:Male; F: Female; Hb: Haemoglobin; Hct: Haematocrit; Ret: Reticulocyte; sPT: Prothrombin time; sAPT: Activated partial thromboplastin time; Cre: Creatinine; Na: Sodium; K: Potassium

	Hb (g/dL)	Hct (L/L)	Ret (%)	WBC (x109/L)	Plt (x109/L)	sPT (secs)	sAPT (secs)	ALT (U/L)	AST (U/L)	CK (U/L)	Urea (mmol/L)	Cre (µmol/L)	Na (mmol/L)	K (mmol/L)	Albumin (g/L)	Globulin (g/L)
**C-1M**	12.9	0.427	1.02	16.61	572	11.8	23.7	34	46	71	7.6	38	145	3.8	41	28
**C-2M**	13.4	0.434	0.92	14.83	410	11.8	25.7	31	35	133	5.12	39	146	4.3	42	24
**C-3M**	12.9	0.447	0.82	13.67	388	10.9	24.2	32	53	59	7.38	41	150	4.2	42	29
**C-1F**	13	0.439	0.7	16.24	384	12.2	25.5	35	37	148	6.54	35	146	4.4	43	26
**C-2F**	12.7	0.419	0.6	15.54	414	11.6	25.5	34	33	112	4.86	48	147	3.5	46	23
**C-3F**	13	0.416	0.76	9.33	452	12.7	24.8	35	33	108	7.52	47	145	3.8	42	26
**LD-1M**	14.4	0.485	0.56	13.48	418	11.4	24.4	28	46	291	8.15	60	147	3.3	42	34
**LD-2M**	12.5	0.408	0.59	11.41	359	11.1	23.1	33	29	91	6.63	51	150	4.3	40	29
**LD-3M**	13.9	0.437	0.5	12.67	493	11.2	23.4	33	29	290	6.52	41	145	3.5	43	30
**LD-1F**	14.5	0.484	0.91	11.69	486	11.6	26.2	21	24	154	5.37	54	147	3.7	46	24
**LD-2F**	14	0.428	0.55	13.52	307	12.2	29.5	26	38	506	6.19	55	146	3.6	43	26
**LD-3F**	13.5	0.423	0.39	10	366	11.4	27	29	29	104	6.69	52	147	4	43	27
**HD-1M**	13.2	0.427	0.87	11.93	504	12.6	23.6	30	31	76	6.22	59	147	3.3	43	27
**HD-2M**	12.8	0.421	0.71	12.21	462	11.1	25.6	46	39	106	6.2	64	149	3.2	47	29
**HD-3M**	12.9	0.422	0.51	10.38	318	12.2	23.1	44	31	82	6.61	54	145	3.9	45	31
**HD-1F**	13.8	0.443	0.78	19.2	408	11.7	23	25	30	88	8.02	53	147	3.8	43	27
**HD-2F**	13	0.425	0.57	14.8	438	11.8	24.1	28	32	230	4.46	66	147	3.7	42	24
**HD-3F**	13.1	0.423	0.42	12.55	293	10.8	22	27	34	68	8.17	64	148	4.3	47	27

Supplementary Table 5. Neutralisation titres against AAVLK03 capsid (1 in X serum dilution). LD: Low dose group; HD: High dose group

Group	ID animal	At selection	Pre-injection	Week 2	Week 4	Week 13	Week 26
Control	10M	Neg	Neg	Neg	Neg	Neg	Neg
	11M	Neg	Neg	Neg	Neg	Neg	Neg
	12M	5	5	5	5	5	5
	15F	5	5	5	5	5	5
	16F	5	5	5	Neg	Neg	Neg
	19F	Neg	Neg	Neg	Neg	Neg	Neg
LD	4M	Neg	5	640	640	640	80
	5M	Neg	Neg	1280	1280	320	160
	6M	Neg	Neg	160	10	Neg	Neg
	13F	Neg	Neg	40	5	5	Neg
	17F	Neg	Neg	640	20	10	Neg
	18F	Neg	Neg	160	40	80	10
HD	7M	Neg	Neg	320	640	1280	160
	8M	Neg	Neg	320	80	80	10
	9M	Neg	Neg	160	40	160	160
	20F	Neg	Neg	40	1280	640	80
	21F	Neg	5	1280	640	640	40
	22F	Neg	Neg	640	20	20	5

Supplementary Table 6. Longitudinal assessment of liver OTC activity. Data of physiological overexpression of OTC activity, OTC activity, and vector genome copy number over time according to experimental group and gender. The increase of OTC activity was calculated as the difference for each animal injected with AAVLK03.hOTC at each time point compared to the average value of OTC activity for control animals of the same gender at the same time point.

Group	ID number	Gender	Increase of OTC activity compared to controls			OTC activity			VGCN		
			Week 1	Week 13	Week 26	Week 1	Week 13	Week 26	Week 1	Week 13	Week 26
Control	10M	Male				52.9	37.8	68.2	0	0	0
	11M					36.2	42.7	34.7	0	0	0
	12M					42.7	56.1	62.0	0	0	0
	15F	Female				36.8	32.3	64.8	0	0	0
	16F					41.3	33.7	45.7	0	0	0
	19F					62.0	36.1	83.0	0	0	0
Low dose	4M	Male	9.0	-8.7	21.9	53.0	36.8	76.8	2.56E+06	2.38E+05	3.34E+05
	5M		5.0	-4.0	10.6	48.9	41.6	65.5	2.69E+06	4.14E+05	4.87E+05
	6M		-7.7	-9.1	7.9	36.2	36.5	62.9	2.71E+06	7.39E+05	9.26E+05
	13F	Female	1.4	17.2	27.1	48.1	51.2	91.6	9.59E+04	8.28E+05	1.10E+06
	17F		4.0	8.6	-5.0	50.7	42.7	59.5	1.58E+06	9.62E+04	1.56E+05
	18F		9.0	0.8	-20.3	55.7	34.8	44.2	2.91E+06	0	0
High dose	7M	Male	5.2	0.0	19.0	49.1	45.5	73.9	3.36E+07	2.04E+07	1.43E+07
	8M		18.9	6.3	5.4	62.9	51.8	60.3	2.69E+07	2.02E+07	1.79E+07
	9M		14.5	8.8	-4.3	58.5	54.3	50.7	4.13E+07	3.24E+07	2.28E+07
	20F	Female	-7.3	15.0	-1.4	39.4	49.0	63.1	2.81E+07	1.04E+07	9.35E+06
	21F		13.7	8.6	-1.9	60.4	42.6	62.6	3.14E+07	1.46E+07	9.83E+06
	22F		-6.9	14.2	25.9	39.8	48.2	90.4	4.02E+07	2.90E+07	2.06E+07

Supplementary information Sequence of codon-optimised human OTC used in AAVLK03.hOTC

ATGCTGTTTAACCTGAGAATCCTGCTGAATAACGCTGCCTTTAGGAACGGACATAACTTCATGGTCCGCAACTTTCGCTGTGGCC
AGCCTCTCCAGAACAAAGTGCAGCTGAAGGGGAGGGACCTGCTGACCCTGAAAAATTTCACAGGAGAGGAAATCAAGTACATGC
TGTGGCTGTCTGCCGATCTGAAGTTCCGGATCAAGCAGAAGGGCGAATATCTGCCACTGCTCCAGGGCAAAAGTCTGGGGATGA
TCTTCGAAAAGAGGAGTACTCGGACCAGACTGTCAACAGAGACTGGATTCGCTCTGCTGGGAGGACACCCATGCTTTCTGACCAC
ACAGGACATTCATCTGGGCGTGAACGAGTCACTGACCGACACAGCCCGGGTGCTGAGCAGCATGGCCGATGCCGTGCTGGCACG
GGTCTACAAACAGAGCGACCTGGATACCCTGGCTAAGGAAGCAAGCATCCCCATCATTAATGGGCTGTCCGACCTGTATCACCCT
ATCCAGATTCTGGCCGATTACCTGACCCTCCAGGAGCATTATTCTAGTCTGAAAGGCCTGACACTGAGCTGGATTGGGGACGGAA
ACAATATCCTGCACTCCATTATGATGTCTGCCGCTAAGTTTGGGATGCACCTCCAGGCAGCCACACCAAAAGGCTACGAACCCGA
TGCCAGTGTGACTAAGCTGGCCGAACAGTATGCTAAAGAGAACGGCACTAAGCTGCTGCTGACCAATGACCCCCTGGAGGCTGC
ACACGGAGGCAACGTCCTGATCACTGATACCTGGATCAGCATGGGCCAGGAGGAAGAGAAGAAGAAGCGGCTCCAGGCCTTCC
AGGGCTACCAGGTGACAATGAAAACTGCCAAGGTCGCCGCTTCTGATTGGACTTTTCTGCATTGTCTGCCCCGAAAACCTGAAGA
GGTGGACGATGAGGTCTTCTATTCACCTAGGAGCCTGGTGTTTCCAGAAGCCGAGAATCGCAAGTGGACAATCATGGCCGTGATG
GTGTCCCTGCTGACTGACTATTCCCCACAGCTGCAGAAGCCTAAGTTTTGA
